# Randomized controlled trial to evaluate the effects of personalized prediction and adaptation tools on treatment outcome in outpatient psychotherapy: study protocol

**DOI:** 10.1186/s12888-017-1464-2

**Published:** 2017-08-24

**Authors:** Wolfgang Lutz, Dirk Zimmermann, Viola N. L. S. Müller, Anne-Katharina Deisenhofer, Julian A. Rubel

**Affiliations:** Clinical Psychology and Psychotherapy, Department of Psychology, University of Trier, D-54286 Trier, Germany

**Keywords:** Precision mental health, Personalized mental health, Routine outcome monitoring, Feedback, Moderators and mediators

## Abstract

**Background:**

Psychotherapy is successful for the majority of patients, but not for every patient. Hence, further knowledge is needed on how treatments should be adapted for those who do not profit or deteriorate. In the last years prediction tools as well as feedback interventions were part of a trend to more personalized approaches in psychotherapy. Research on psychometric prediction and feedback into ongoing treatment has the potential to enhance treatment outcomes, especially for patients with an increased risk of treatment failure or drop-out.

**Methods/design:**

The research project investigates in a randomized controlled trial the effectiveness as well as moderating and mediating factors of psychometric feedback to therapists. In the intended study a total of 423 patients, who applied for a cognitive-behavioral therapy at the psychotherapy clinic of the University Trier and suffer from a depressive and/or an anxiety disorder (SCID interviews), will be included. The patients will be randomly assigned either to one therapist as well as to one of two intervention groups (CG, IG2). An additional intervention group (IG1) will be generated from an existing archival data set via propensity score matching. Patients of the control group (CG; *n* = 85) will be monitored concerning psychological impairment but therapists will not be provided with any feedback about the patients assessments. In both intervention groups (IG1: *n* = 169; IG2: *n* = 169) the therapists are provided with feedback about the patients self-evaluation in a computerized feedback portal. Therapists of the IG2 will additionally be provided with clinical support tools, which will be developed in this project, on the basis of existing systems. Therapists will also be provided with a personalized treatment recommendation based on similar patients (Nearest Neighbors) at the beginning of treatment. Besides the general effectiveness of feedback and the clinical support tools for negatively developing patients, further mediating and moderating variables on this feedback effect should be examined: treatment length, frequency of feedback use, therapist effects, therapist’s experience, attitude towards feedback as well as congruence of therapist’s and patient’s evaluation concerning the progress. Additional procedures will be implemented to assess treatment adherence as well as the reliability of diagnosis and to include it into the analyses.

**Discussion:**

The current trial tests a comprehensive feedback system which combines precision mental health predictions with routine outcome monitoring and feedback tools in routine outpatient psychotherapy. It also adds to previous feedback research a stricter design by investigating another repeated measurement CG as well as a stricter control of treatment integrity. It also includes a structured clinical interview (SCID) and controls for comorbidity (within depression and anxiety). This study also investigates moderators (attitudes towards, use of the feedback system, diagnoses) and mediators (therapists’ awareness of negative change and treatment length) in one study.

**Trial registration:**

Current Controlled Trials NCT03107845. Registered 30 March 2017.

## Background

Psychotherapy is successful for the majority of patients, but a substantial proportion of the patient population does not improve or even deteriorates during treatment. Evidence suggests that between 5% and 10% of the patients leave treatment worse off than before treatment [1]. In the future, the outcome of psychological interventions could be enhanced by empirical recommendations regarding the most promising treatment strategies for a given patient (personalized predictions); as well as supplementing the traditional treatment modalities with ongoing outcome measurement and feedback or problem solving tools (personalized adaptations; [2–5]). Such prediction and treatment adaptation tools can and will likely be more easily implemented by using tools from eMental health research [6]. The more technology develops, the easier it is to implement these tools into routine care. The recent debate about precision and personalized medicine is reflected in the different purposes of such tools. While prediction tools can be seen as part of precision mental health, which tries to forecast the most promising treatment approach or strategy given specific patient characteristics, adaptation tools can be seen as part of a personalized mental health approach in which ongoing treatments are tailored to the individual patients treatment course. So far such prediction and adaptation or feedback tools have been studied independent of each other [3, 5]. However, in order to maximize the benefits of both approaches they plausibly need to be combined in a comprehensive model. The herein described trial tests such a tool which combines these two approaches in order to provide therapists with recommendations before the treatment and throughout especially for patients at risk for treatment failure.

Precision mental health has only recently received considerable attention. For example, a new method has been introduced, which aims at treatment selection based on empirical data, namely the Personalized Advantage Index (PAI; [3, 4]). Using multiple regression methods that weigh the predictive value of different patient intake characteristics, the PAI is a measure of the potential advantage of a Treatment A over a Treatment B. The use of the PAI has been shown in two applications: In the first demonstration, DeRubeis et al. used the PAI to predict which patients would profit more from CBT than an antidepressive medication (ADM) and vice versa [3]. In the second study, Huibers et al. demonstrated the PAI’s potential for the selection between cognitive therapy (CT) and IPT [4]. Another methodology was adapted for the prediction of treatment response by Lutz et al. in a sample of 618 psychotherapy outpatients [2]. In accordance with avalanche prediction models (e.g. [7]), the response curves of the most similar patients who had already been treated were used to derive a prediction for a newly incoming patient. Similarity among patients was defined in terms of Euclidean distances between the relevant predictor variables, which was also called the nearest neighbor approach (NN). The authors tested the predictive validity and clinical utility of the NN approach for treatment selection: For each patient, the authors generated predictions for two treatment protocols (CBT vs. an integrative CBT and interpersonal treatment [IPT] protocol) and compared whether one of these treatments was predicted to be more or less beneficial for a specific patient. Although, on average, no significant outcome difference between the two protocols was found, with the NN method, it was possible to obtain clinically meaningful differential outcome predictions for about one third of the patients. For the other two-thirds, the predicted change did not differ between the two protocols [2].

So far, precision mental health predictions have been only tested in post-hoc analyses. No study thus far has applied these predictions in a prospective trial in which they are provided to clinicians.

More studies have been conducted regarding adaptation of ongoing treatments based on routine outcome monitoring and feedback tools. Several international research groups investigated such tools (mostly feedback systems) in randomized controlled trials (RCT). The first three RCTs in that field found that feedback to therapists on patients’ progress was effective in improving patient outcomes, particularly for those patients who showed an increased risk for treatment failure (not on track patients; NOT). The percentage of patients at risk for treatment failure receiving feedback and reaching a reliable or clinical significant improvement was about 14% higher than the rate for the NOT patients without feedback [8–10]. Furthermore, those NOT patients with feedback had an 8% lower deterioration rate than without feedback. Additionally, feedback to therapists of NOT patients led to, on average, longer treatments. However, it led to shorter treatments, on average, for on-track (OT) patients. Those early investigators came to believe that the essential value of feedback systems was to help clinicians become aware of pending treatment failure, something that they could not achieve through clinical intuition [11].

To date, key findings of these original studies have been replicated under different conditions in many investigations and have been reported in four systematic reviews [12–15] and five meta-analyses [16–20]. For example, in a recent review on the effects of feedback Krägeloh and colleagues (2015) report that of the 25 identified studies, 17 showed a significantly positive feedback effect on average or for NOT patients [14]. More detailed information about the size of the effects derive from several meta-analyses [17, 18, 20]. In these studies the significant effect sizes regarding improvement for NOT patients with feedback vs. treatment as usual (TAU) varied between an effect size of g = .22 and g = .53. However, a recent Cochrane report graded the quality of evidence for these effects as low and requests further studies with stronger designs [20]. When clinical support or problem solving tools (CST) were implemented the effect size for NOT patients increased and reached g = .70 [18]. CST’s help to determine the potential cause of deterioration and provide clinical suggestions to adapt or improve treatments at risk for failure.

From this cumulative body of research it can be concluded that feedback is effective for NOT patients especially in combination with CSTs. However, most feedback studies were conducted within settings with relatively short treatments provided to moderately impaired patients (e.g., college counseling centers; [15, 18, 19]. Recent studies investigated feedback effects in more disturbed outpatients [21, 22], in psychosomatic in-patients [23, 24], patients with eating disorders, PTSD, patients with substance use disorders, depression and anxiety disorders [21–25]. No study so far has investigated diagnostic group as a moderator of the feedback effect.

De Jong et al. [21] found substantial differences between therapists in their use of feedback. Having a higher commitment to use the feedback showed to be significantly associated with a higher probability to use the feedback and therapists who indicated to use the feedback showed to be more effective for patients with a risk of treatment failure (NOT patients). Additionally, therapists who were more committed to use the feedback at the beginning of the study had patients who progressed faster in treatment. Similarly, Knaup et al. [16] found in a meta-analysis of 12 feedback studies that the frequency of feedback given in the studies as well as the kind of feedback (progress vs. status) seem to be promising moderators. Furthermore, Lutz, Rubel, Schiefele, Zimmermann, Böhnke, and Wittmann [26] found that patients’ as well as therapists’ attitudes towards feedback combined with the use of the feedback system was significantly associated with treatment outcome. Those therapists, which had a positive attitude related the feedback system and did respond with specific actions did show the best effect sizes, whereas therapists with a negative attidude but many, probably uncoordinated, actions had the worst effect sizes.

Treatment length is the most studied mediator variable. In this context one finding was that patients with negative feedback (NOT) stayed longer in treatment while patients with positive feedback (on track; OT) had fewer sessions in comparison to therapies where no feedback was provided [27]. This finding suggests that the effects of feedback on treatment outcome might be mediated by treatment duration. However, the effect of feedback on number of sessions was not consistently found [16, 20, 23, 28]. Therefore, a further investigation whether feedback influences the length of treatments and in which way this might be connected to the effects of feedback on outcome is needed.

In summary, the described studies on psychometric feedback have specific strengths and weaknesses. While in most RCTs the high frequency of repeated measurements in the feedback as well as in the control conditions can be evaluated positively, studies often included treatments with few sessions and moderately impaired patients [19]. Further weaknesses are that past studies did not include structured diagnosis or the control of treatment adherence. Also most studies were restricted to commercial feedback systems and did not include prediction or treatment selection tools.

Furthermore, above described moderators and mediators were found with post-hoc analyses as a by-product of explorative analyses in different studies. No specific power analyses was conducted to study these effects and they have not been studied in a comprehensive model within the same study.

## Objectives

### Primary research questions and hypotheses

The primary objective of the current project is to investigate several questions of personalized psychotherapy research with two personalized feedback intervention groups (IG1: Feedback; IG2: Feedback plus CSTs (including personalized predictions before the start of the treatment and adaptation tools during treatment) and one control group (CG) with repeated measurements. As such, this is the first study that prospectively tests the effects of personalized treatment recommendations and adaptation tools. Moreover, it adds to previous feedback research a stricter design by investigating another repeated measurement CG as well as a stricter control of treatment integrity. It also includes a structured clinical interview (SCID) and controls for comorbidity (within depression and anxiety disorders). Furthermore, a severely impaired patient sample is studied and an international outcome instrument is used. This study also investigates the above described moderators (attitudes towards, use of the feedback system, diagnoses) and mediators (therapists’ awareness of negative change and treatment length) in a comprehensive model and in one study.

**Fig. 1:**
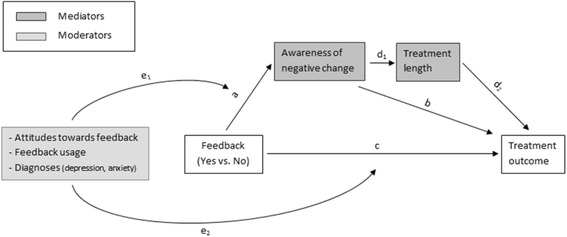
The mediating and moderating role of variables in the effect of feedback on treatment outcome (path c)

Therefore, the study allows for testing the following hypotheses:

- Main hypotheses:
  - H1: *NOT patients in the feedback condition (IG1) show on average better treatment outcomes than NOT patients in the CG.*
  - H2: *NOT patients in the IG2 (+personalized prediction and adaptation tools) show on average better treatment outcomes than NOT patients of the IG1 (no tools but psychometric feedback).*
- Secondary hypotheses concerning moderators and mediators (see also Fig. 1):
  - H3: *The positive impact of feedback for NOT patients is* moderated *through the usage of the feedback system and/or the attitudes of the therapist towards feedback.*
    - *H3a: The more frequently and the longer feedback is used, the more aware are therapists for negative change.*
    - *H3b: The more positive the therapists’ attitudes towards feedback, the more aware are therapists for negative change.*
    - *H3c: The effects of feedback on patient outcomes do not differ between diagnostic groups (depression and anxiety).*
  - H4: *The positive impact of feedback for NOT patients is* mediated *through therapists’ awareness of negative change as well as treatment length.*
    - *H4a: The positive impact of feedback for NOT patients is mediated by the therapists’ awareness of negative developments of their patients.*
    - *H4b: The positive effect of therapists’ awareness on treatment outcome is mediated by treatment length (number of sessions).*

## Methods

### Study design

This study is a partially randomized controlled trial. Clients will be randomly assigned to one of two groups. An additional intervention group (IG 1) will be generated from an existing archival data set via propensity score matching.

1. Control group (CG): Treatment with continuous assessments but without computer based feedback to therapists.
2. Intervention group 1 (IG1): Patients in this group received treatment with continuous assessments including computer-based feedback to therapists after each session. This group is a matched sample.
3. Intervention group 2 (IG2): Treatment with continuous assessments including computer based prediction as well as feedback/adaptation tools to therapists after each session including an alarm for NOT patients.

The project was submitted to and approved by the Ethics Committee of the University of Trier. Since it is a non-invasive procedure, patients are informed about data protection law consequences and their opportunity to refuse to accept the storage and use of video data for research purposes at any time. An explanation of the various experimental conditions will be given at the end of the project.

### Setting

The patient samples will be assessed at the research outpatient clinic at the University of Trier. Treatments are conducted by cognitive-behavioral therapists in training with different levels of experience. In the outpatient clinic standardized ways of recording and documenting treatment data is established. Session reports are assessed via touch screen data entry devices, whereas pre- and post as well as 5-session assessment are entered by research assistants. The infrastructure has been further improved for this study. Computerized status and progress feedback is provided via a secured website (feedback portal).

### Inclusion and exclusion criteria

The sample will include all patients who enter psychotherapy during the recruitment period and fulfill the following inclusion criteria:

- At least one anxiety or depressive disorder (ICD-10: F32, F33, F40, F41, F42, F43)
- At least 3 treatment sessions

The exclusion criteria are:

- Organic, including symptomatic mental disorders (ICD-10: F00-F09)
- Mental and behavioral disorders due to psychoactive substances (ICD-10: F10-F19)
- Schizophrenia, schizotypal, and delusional disorders (ICD-10: F20-F29)
- Acute suicidality

### Randomization

**Fig. 2:**
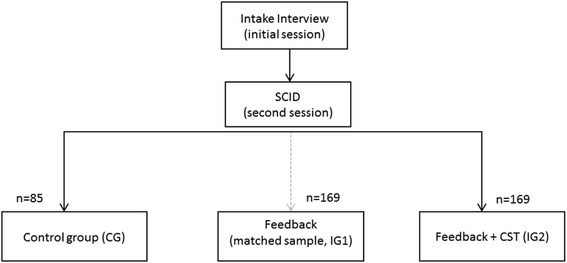
Diagram of patient flow within the study

First, patients will be randomly assigned to therapists after the SCID-I interview which will be conducted by an independent trainee (see Fig. 2). Second, patients will be randomized to one of two groups (CG or IG2). This means that the same therapist treats patients who are in the CG as well as patients who are in the IG2. The assignment procedure of patients to therapists will secure, that each therapist will treat at least eight patients and that each therapist has patients in both conditions. As an additional randomization condition a matching procedure is applied, which ensures that the therapists do not differ in terms of their level of clinical experience and years of using the feedback system in IGs and the CG. A second intervention group (IG1) is generated via propensity score matching from already existing archival data set based on diagnoses, intake severity as well as social-demographic variables like age and gender.

### Assessments

Table 1 gives an overview of the instruments as well as their time of measurement within the project.

**Table 1:** Diagnostic instruments and assessment schedule

	Begin	Every session	Every 5th session	End
SCID-Interview [29] (SCID)	x			
Brief Symptom Inventory [33] (BSI)	x			x
Outcome Questionnaire [53] (OQ-30)	x		x	x
11-item Short version of Hopkins‘Symptomchecklist [31] (HSCL-11)		x		
Assessment for Signal Clients [5] (ASC)	x		x	
Affective Style Questionnaire [54] (ASQ)	x		x	
Patient Health Questionnaire [55] (PHQ-9)	x		x	
Generalized Anxiety Disorder [56] (GAD-7)	x		x	
Global Assessment of Functioning [41] (GAF)	x	x		x

In addition to psychometric instruments, socio-demographic and psychosocial data of both patient and therapist (e.g. clinical experience) are collected as part of the basic documentation system in the outpatient center. Axis I diagnoses are assessed with the SCID-I [29] and Axis II disorders are assessed with the IDCL-P checklist [30]. Both assessment procedures are part of the already existing routine within the outpatient center.

### Assessment of outcome instruments

#### Hopkins symptom checklist – short form (HSCL-11)

The HSCL-11 is an 11-item self-report inventory for the assessment of symptomatic distress [31]. It was developed based on the HSCL-25, which is a brief version of the Hopkins Symptom Checklist-90 [32]. In the present study, the HSCL-11 will be administered at the beginning of each session. The items are responded to on a 4-point Likert scale ranging from 1 (“not at all”) to 4 (“extremely”). The mean of the 11 items represents the patient’s level of global symptomatic distress, it is highly correlated with the GSI (*r* = 0.91) and also has high internal consistency (α = .92; [31]).

#### Brief symptom inventory (BSI)

Symptom severity will be measured pre- and post treatment using the BSI ([33]; German translation of [34]) which is a 53-item self-report inventory inquiring about physical and psychological symptoms within the last week. It is the brief form of the Derogatis’ Symptom Check-List-90 Revised (SCL-90-R; [34]), which assesses 9 subscales with the following dimensions: somatization, obsessive-compulsive, interpersonal sensitivity, depression, anxiety, hostility, phobic anxiety, paranoid ideation, and psychoticism. Item response takes place on a 5-point Likert scale ranging from 0 (“not at all”) to 4 (“extremely”). Psychometric properties for this index can be regarded as excellent (αpre = .96; αpost = .97).

#### Outcome questionnaire-30 (OQ-30)

The OQ-30 will be administered pre-; each 5 sessions and post-treatment. This 30 items self-report measure is designed to assess patient outcomes during the course of therapy. The OQ has three primary dimensions: (a) subjective discomfort, (b) interpersonal relationships, and (c) social role performance. All 30 items can be aggregated to create a total score. The OQ-30 is a short from of the OQ-45 comprising the 30 items that are most sensitive to client change and demonstrated high levels of congruence with the OQ-45 in measurement of patient outcome [35–37]. The OQ-30 showed an adequate internal consistency in our sample (α = .90). For an enhanced comparability the OQ-30 will be applied because it is internationally the most widely used instrument in feedback studies [12].

As part of the feedback and adaptation tools (see below), the ASC and ASQ are assessed every fifth session. To be able to compare the results of this project to feedback studies conducted in the UK [38] the GAD-7 and PHQ-9 will be used as symptom specific instruments every fifth session.

### Assessment of feedback related variables

To examine the usage of the feedback system, user statistics for each therapist will be recorded [39, 40]. These access statistics of the feedback system will allow to evaluate the frequency of feedback use as well as the amount of time spend within the feedback system and for each specific case.

Therapists’ attitudes towards feedback will be assessed as soon as each therapist starts working within the project. In order to evaluate the modifications therapists make due to feedback (Fig. 1, path b), therapists will be asked ten specific questions after the termination of each treatment.^1^ For control purposes therapists in the control group will also be asked whether they used additional questionnaires or some material from the clinical support tools even though this material was not available for this patient (but for a different patient of this therapist).

### Assessment of therapists’ awareness of negative change

In order to evaluate therapists’ awareness of patient negative change, the congruence between therapist and patient estimates of outcome is calculated, when the patient is NOT. Therefore, therapist rated outcome will be assessed at each session via the Global Assessment of Functioning Scale (GAF; [41]). In accordance with the truth and bias model, congruence is defined as the correlation between the therapists’ assessments of patients’ global functioning (measured with the GAF) and patients’ assessment of their functioning (measured with the HSCL) [42, 43]. In this sense, the more similar therapists’ change ratings are in relation to patients’ change ratings, the better is the awareness of the therapists concerning the negative change of this specific patient.

### Assessment of control variables

Treatment integrity will be assessed as adherence and competence. Competence will be evaluated with the Cognitive Therapy Scale (CTS; [44, 45]) and adherence with the Cognitive-Behavioral Therapy Adherence Scale (CBT-AS; [46]). All sessions are videotaped. Three master-level independent raters will evaluate a random selection of 10% of all sessions per treatment to assess the quality of cognitive therapy. All raters will be trained in an 18-h training prior to the evaluation and will be blinded concerning treatment outcome and treatment condition. Also interrater reliability will be ensured in the training procedure. To check for the use and influence of non-psychometric feedback on the effects of psychometric feedback the item 8^2^ in the CTS scale will be used as a control variable (using feedback and summaries).

### Implementation of feedback and clinical adaptation and support tools

**Fig. 3:**
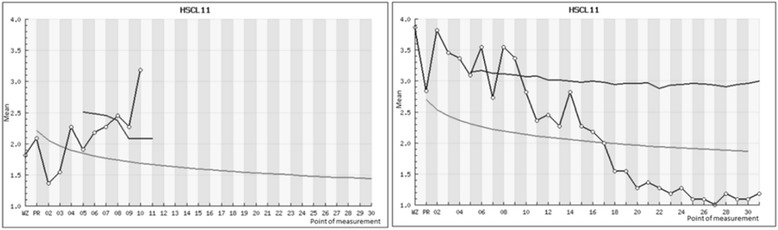
Progress of two different simulated patient examples measured with the HSCL-11. On the left, a negatively developing patient who went off-track during the course of treatment and on the right side a positively developing patient who stays on-track after session 10

Each therapist is able to login after each session to the online feedback portal to get an overview about status and progress of his/her patient in the intervention groups (IG1 & IG2). For patients of the IG1, therapists are provided with information about the initial status concerning symptoms (BSI & OQ-30), interpersonal functioning (IIP-32 & OQ-30) as well as diagnoses specific symptoms (GAD-7 or PHQ-9). Beside the status measures, also individual progress information on the symptom level (HSCL-11 each session) is provided to the therapist (see line with dots in Fig. 3). Higher values indicate more distress. The gray curve represents the expected treatment response for this specific case based on growth curve models developed for the nearest cases (nearest neighbors). Additionally to the expected treatment response (gray curve) a black signal curve beginning from session 5 is displayed (90% confidence boundary). This curve will be recalculated after each session considering the already made progress up to this point. When a patient lies above the black curve (90% confidence boundary), a warning signal is presented within the feedback system.

**Fig. 4:**
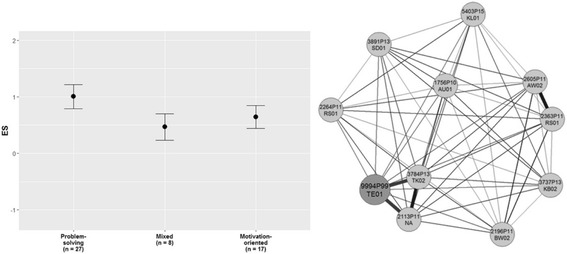
Personalized strategy recommendations and nearest neighbor therapists

**Fig. 5:**
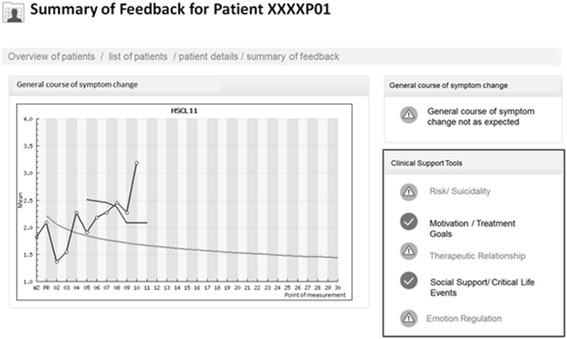
Example screenshot from the feedback system in the IG2. All orange signals within the box are linked to respective adaptation and problem solving tools. For therapists in the IG1 neither the signals in the red box nor the access to the tools will be included

Therapists of the IG1 will be able to see status and progress reports as described above (including an overall evaluation of progress in the HSCL, but not expected treatment response and the confidence boundary) whereas the IG2 will get additional access to CSTs implemented in the system. The CSTs are divided into two main areas: a personalized treatment recommendation module and a personalized treatment adaptation module.

### Personalized treatment recommendation

The personalized treatment recommendation is available at the beginning of treatment. The therapists will be provided with important information for their patients on different domains like risk for suicidality, substance abuse, treatment expectation, drop-out risk, symptomatology, and interpersonal functioning.

Additionally, a treatment recommendation for the early phase of treatment is given (Fig. 4). Already treated similar cases within the outpatient center are selected. Therapists rated whether they chose a more problem-solving focused approach, a more motivation-oriented approach or a mixed approach for these cases based on session reports. Based on the closest cases of already treated patients with those three strategies an effect-size is calculated for each of the three interventions. Thus, therapists receive a recommendation which early treatment strategy might lead to the best treatment effect for this particular case. The ten closest cases are depicted in another graph with their treating therapist. This leads to the opportunity that therapists are able to contact therapists who already treated similar cases for peer-supervision.

### Personalized treatment adaptation

To make sure that adaptation and support tools are addressing the right domains in the course of treatment, the Assessment for Signal Clients (ASC) as well as the Affective Style Questionnaire (ASQ) are measured every fifth session (Fig. 5). The four subscales of the ASC are used to measure therapeutic alliance, motivation, social support, and life events. Based on a cut-off value for each measure “orange” (in Fig. 5 light grey) or “green” (in Fig. 5 dark grey) signals are provided for patients who were identified as on risk for treatment failure. The ASQ is used as a marker for the extent of the emotional regulation ability of the patient with the same purpose. If orange signals are detected on one of the five scales (ASQ and/or ASC) then the therapist is able to click on the scale and to open a website with more information on those tools for a specific domain. This additional specific clinical guidelines are oriented on the Clinical Support Tools developed by Lambert et al. and are translated and adapted to the German outpatient setup [5]. Those original tools are extended by using additional clinical as well as video and audio material. Prior to the beginning of the project, participating therapists are instructed and trained on the new feedback portal by the research associate of the project and a video clip, which explains the system.

### Sample size calculation and data analyses

Since the two main hypotheses are based on pairwise comparisons of the NOT patients, the sample size calculation was done so that the size of these comparisons have sufficient power (1-β = .8). Power analyses for the main hypotheses have been conducted with GPower 3.1 [47]. Hypothesis 1 postulates a superior treatment outcome for NOT patients in the IG1 compared to the CG. The average effect for feedback in NOT patients compared to treatment as usual found in the literature is between *r =* .25 [17]. To find this effect in a two-way repeated measures ANOVA with (feedback yes/no) and assessment time (pre/post) with a power of at least 80%, an a priori significance level of .05, and a correlation between assessments of .4, the sample has to include at least *N* = 38 NOT patients in total (IG1 and CG). The incremental effect for feedback with CSTs in comparison with feedback without CSTs was *d =* .31 [48]. We therefore assume a small incremental effect (*d =* .30) for the use of CST in this project. To find this effect in a two-way ANOVA with (feedback yes/no) and assessment time (pre/post) with a power of at least 80%, an a priori significance level of .05, and a correlation between assessments of .4, the total sample has to include *N* = 108 NOT patients in total (IG 1 and IG 2). To make sure, that treatment groups and CST are more comparable with respect to the number of patients, we added for the sample size calculation in the CG half of the NOT patients in IG1/IG2, resulting in at least 27 NOT patients for the CG.

In a previous study the proportion of patients who received at least one alarm signal within the first 8 sessions was approximately 49% [49]. Also, Simon et al. found that 56% of the patients were NOT and therefore received an alarm signal [22]. For our sample size calculation we used a more conservative estimate of 40% of NOT patients, which results in a total sample size of *N* = 338 (CG = 68, IG = 135, IG2 = 135). In order to control for drop-out, we added a puffer of 25% to the required sample size to make sure the power analysis is valid. The described procedure above, results in final sample sizes for each group shown in Table 2.

**Table 2:** Intended recruitment and sample size of treatment groups

Treatment	Sample size
Intervention group 1	169
Intervention group 2	169
Control group	85

Three moderators and two mediators of the feedback effect will be investigated in hypotheses 3 and 4 (see also Fig. 1): Moderators: therapists’ attitudes towards feedback, feedback usage and diagnoses; Mediators: Therapists’ awareness for negative developments and treatment length. In order to control for therapist effects, therapists will be modelled as random effects at level two.

Therefore, *Optimal Design Software for Multilevel and Longitudinal Research* (Version 3.01; [50]) was used to determine the minimal effect that can be detected with sufficient power, given the sample size calculation above. Assuming a therapist effect of 5% and k = 50 therapists delivering the treatments to j = 8 patients each, a small effect (d = .2) can be detected with sufficient power (1-β = .8). Thus, given the sample size calculated for the primary hypotheses, the secondary analyses can be conducted with sufficient power using multi-level models (e.g., [51]). Ensuring the stability of the results, all analyses will be checked with Mplus software (e.g., [52]).

Additional Monte Carlo simulations with Mplus (Version 7; [52]) were run to specifically test the mediation models from hypotheses 3 and 4. Assuming again small effects for the direct associations, 10,000 datasets have been simulated with the actual sample size of the study sample. These simulations revealed a sufficient power of 1-β = .874. Thus, given the sample size calculated for the primary hypotheses, the mediation analyses can also be conducted with sufficient statistical power to detect indirect mediation effects adjusting for above described control variables.

Furthermore, the analyses will be controlled for the following potential influencing factors: comorbidity (number of additional diagnoses, additional personality disorders), initial impairment, clinical experience as well as experience with the feedback system.

Finally, the data of the project will have a hierarchical data structure because patients will be nested within therapists. Two-level hierarchical models will be applied to the data to correct for therapist influences [51].

## Discussion

The present study protocol describes the implementation of a cluster randomized controlled trial to investigate several questions of personalized and feedback research in psychotherapy. The primary objective of the project is to examine the effects of psychometric prediction and adaptation tools on treatment outcome within an outpatient clinic under routine care conditions. The aim is to use empirical data and prediction and problem solving tools to support clinical decisions at the beginning as well as during the course of treatment. Furthermore, this is the first study to include potential mediators and moderators of the feedback effect in one study design to replicate findings that have been investigated so far only in single studies. In this way, the study does not only enhance the existing literature by showing whether feedback could improve therapy. It also helps in understanding the underlying mechanisms of action.

Furthermore, there are a number of aspects to this study that have rarely been brought together in feedback research and which will add valuable evidence to the existing body of research. This comprises the investigation of feedback in the context of longer treatments, severely impaired patients and structured diagnoses. Above this, the study will focus on treatment integrity in assessing adherence and competence in a structured way. Former studies did not include this aspect. Besides this, the second goal of the project is the development of a public domain software based on an already existing feedback system used in the outpatient center at the University of Trier. So far, the software includes options for data collection, data management and basic feedback. Within the study we will expand the software with advanced prediction tools as well as an elaborated psychometric feedback including clinical support and problem solving tools for therapists. The adaption of the software is part of the research project which will lead to a cost-free software under a GNU General Public License open to interested research groups after the end of the project.

## Trial status

Currently recruiting (N_current_ = 116 as of June 2017).

## Footnotes

1 Ten specific questions will be asked e.g., “Due to feedback, I…”

- …discussed with the patient his/her answers in the questionnaire
- …tried to adjust my therapeutic interventions
- …prepared the end of therapy
- …etc. (for more details see Lutz et al., 2012)

2 The therapist should ask for feedback regularly to ensure his or her own understanding of the patient’s situation and the patient’s understanding of therapy. The therapist should acquire feedback in such a way that the patient does not feel tested or evaluated.
